# The First Description of the Microbial Diversity in the Amarillo River (La Rioja, Argentina), a Natural Extreme Environment Where the Whole Microbial Community Paints the Landscape Yellow

**DOI:** 10.3390/microorganisms12020235

**Published:** 2024-01-23

**Authors:** Cecilia Elena Bernardelli, Deborah Colman, Edgardo Ruben Donati, María Sofía Urbieta

**Affiliations:** Centro de Investigación y Desarrollo en Fermentaciones Industriales (CINDEFI), Consejo Nacional de Investigaciones Científicas y Técnicas (CONICET), 50 N 288, Calle, La Plata 1900, Buenos Aires, Argentina; cecibe@quimica.unlp.edu.ar (C.E.B.); debocolman@googlemail.com (D.C.); donati@quimica.unlp.edu.ar (E.R.D.)

**Keywords:** iron cycle, acidic environment, microbial interactions, geomicrobiology

## Abstract

The Amarillo River in Famatina, La Rioja, Argentina, is a natural acidic river with distinctive yellow-ochreous iron precipitates along its course. While mining activities have occurred in the area, the river’s natural acidity is influenced by environmental factors beyond mineralogy, where microbial species have a crucial role. Although iron-oxidising bacteria have been identified, a comprehensive analysis of the entire microbial community in this extreme environment has not yet been conducted. In this study, we employ high-throughput sequencing to explore the bacterial and fungal diversity in the Amarillo River and Cueva de Pérez terraces, considered prehistoric analogues of the current river basin. Fe(II)-enrichment cultures mimicking different environmental conditions of the river were also analysed to better understand the roles of prokaryotes and fungi in iron oxidation processes. Additionally, we investigate the ecological relationships between bacteria and fungi using co-occurrence and network analysis. Our findings reveal a diverse bacterial community in the river and terraces, including uncultured species affiliated with Acidimicrobiia, part of an uncharacterised universal microbial acidic diversity. Acidophiles such as *Acidithiobacillus ferrivorans*, the main iron oxidiser of the system, and *Acidiphilium*, which is unable to catalyse Fe(II) oxidation but has a great metabolic flexibility,, are part of the core of the microbial community, showing significant involvement in intraspecies interactions. *Alicyclobacillus*, which is the main Fe(II) oxidiser in the enrichment culture at 30 °C and is detected all over the system, highlights its flexibility towards the iron cycle. The prevalence of key microorganisms in both rivers and terraces implies their enduring contribution to the iron cycle as well as in shaping the iconic yellow landscape of the Amarillo River. In conclusion, this study enhances our understanding of microbial involvement in iron mineral precipitation, emphasising the collaborative efforts of bacteria and fungi as fundamental geological agents in the Amarillo River.

## 1. Introduction

Acidic environments are defined by pH values lower than 3 in their aqueous media; nevertheless, they are generally poli-extreme environments, where, besides pH, other physicochemical parameters are beyond the human tolerable range, for instance, high temperatures in geothermal areas, high pressures in hydrothermal vents and high metal concentrations in sulphur ore deposits [1]. Indeed, acid mine drainages are among the most studied acidic environments due to their negative impact on soil and water courses and eventually on animals and human life, caused by the mismanagement of mining activity. A less explored variant is natural acidic environments, which have similar extreme conditions but are merely associated with sulphur mineralogy or volcanic activity, such as the famous Rio Tinto (Huelva, Spain) or Río Agrio (Neuquén, Argentina) [2,3]. Despite the harsh physicochemical conditions, in addition to the limitations in energy and carbon sources imposed by sulphur minerals, the biodiversity of AMD-related environments is far from being delimited to a handful of well-adapted species as would have been originally expected. Since the breakthrough of high-throughput omic techniques, knowledge on this subject has expanded enormously with the constant appearance of novel taxa of bacteria and archaea ([4,5]), which alone justifies further study, particularly of novel, unexplored, natural acidic environments.

The Amarillo River which runs for 35 Km along the eastern slope of the “Nevado de Famatina” hill (its actual name is General Belgrano hill) in La Rioja province, Argentina, is a perfect case of a novel, unexplored, natural acidic environment. The headwaters of the river drain an important alteration halo with multiple porphyry-type Cu (Mo-Au) deposits and high sulfidation Cu-Au (Ag-As-Sb-Te) veins; the most abundant mineral is pyrite (FeS_2_), associated with enargite (Cu_3_AsS_4_), famatinite (Cu_3_SbS_4_), tetrahedrite-tennantite (Cu_12_As4S_13_-Cu_12_Sb4S_13_), chalcopyrite (CuFeS_2_), sphalerite (ZnS) and galena (PbS) (among others) as subordinate phases [6]. The area presents a dry continental high mountain climate (maximum altitude of approximately 6000 m.a.s.l.), with about 200 mm per year of rainfall, mainly between January and March and infrequent snowfall during the winter. The temperature ranges from 24.7 °C (summer) to 8.5 °C (winter), with an annual average of 17.1 °C.

Due to the geology described, the Amarillo River is characterised by turbulent acidic sulphate waters, with a pH close to 3 for most of its course, high concentration of heavy metals and metalloids such as Fe, Mn, As, Cu, Zn, Pb, Co, Cr, Ni, Cd and Mg, among others, and high total dissolved solids. Along the course of the river, there are widespread deposits of ochre sediments associated with the precipitation of Fe(III) compounds. The main phase of these precipitates found in the upper section of the river belongs to minerals from the group of jarosite (XFe_3_(SO_4_)_2_(OH)_6_), while schwertmannite (Fe_8_O_8_(OH)_6_(SO_4_)·nH_2_O) dominates the middle and lower sections [7]. 

Based on the analysis of the sediments and waters of the Amarillo River, a purely geochemical model was presented by Maza and co-workers [8], which was later reformulated considering only the characteristics of certain microbial species found in enrichment cultures obtained from the river sediment samples [9]. Briefly, the model proposed that the oxidative dissolution of pyrite and other sulphides starts at the head of the river, which generates acidic and oxidising conditions that lixiviate insoluble elements, consequently increasing the concentration of Fe, mainly in the ferrous form, as well as Zn, Cu and As. The abiotic oxidation of Fe(II), which is very slow at the environmental conditions of the Amarillo River [10], is catalysed by iron-oxidising bacteria, such as *Acidithiobacillus ferrivorans* and *Leptospirillum ferrooxidans*, generating higher concentrations of the powerful oxidising agent Fe(III).

On the other hand, mycodiversity in extreme environments and their role in biogeochemical processes is scarcely considered, even though fungi are remarkably capable of adapting and surviving in a great variety of environmental conditions due to their morphological, physiological and ecological versatility, including symbiosis, parasitism, saprophytic nutrition, sexual and asexual reproduction and spore formation, to name only some adaptative strategies ([11,12]). Particularly in acidic environments, there are only a few assessments of eukaryotic biodiversity in Rio Tinto, acid mine drainages and acidic soils ([13,14,15]), and only a few isolated acidophilic fungal species have been reported [16]. For instance, the acidophilic fungi *Purpureocillium lilacinum* has been associated with the precipitation of jarosite in Río Tinto [17]. It also has been described that fungi are important in the formation and structure of biofilms in acidic environments and that they might act as primary decomposers in the absence of invertebrates [13]. However, fungal diversity, the characterisation of acidophilic fungal species and their correlation with prokaryotic diversity and environmental parameters are hardly ever included in studies on the ecology of acidic environments.

Understanding the geomicrobiology and biodiversity of acidic environments is essential for unravelling the mechanisms by which microorganisms adapt to and contribute to the biogeochemical cycling of elements in these extreme conditions. While acidic mine environments have been extensively studied, there is a notable scarcity of research on naturally acidic settings. Investigating such environments offers valuable insights into the intrinsic capabilities of microorganisms to thrive in extreme acidity, unperturbed by anthropogenic factors. This broader exploration of acidic environments contributes to a comprehensive understanding of microbial life across diverse ecological niches and provides a foundation for broader implications in fields ranging from environmental science to biotechnology.

In this context, the aim of this work is to reveal the prokaryotic and fungal microbial community of the Amarillo River and its terraces to understand their importance in the geomicrobiological processes occurring in this natural extreme environment. Moreover, to further understand the role of the microbial species associated with iron precipitate formation, we also studied the microbial consortia obtained from river samples cultured at pH, temperature and nutritional conditions representative of the different stages of the Amarillo River and/or optimum for the development of different members of the community that could be involved in the iron cycle. The approach was carried out using, for the first time in this system, high-throughput sequencing technology that supported co-occurrence and network interaction analysis.

## 2. Materials and Methods

### 2.1. Site Description and Sample Collection

**Figure 1:**
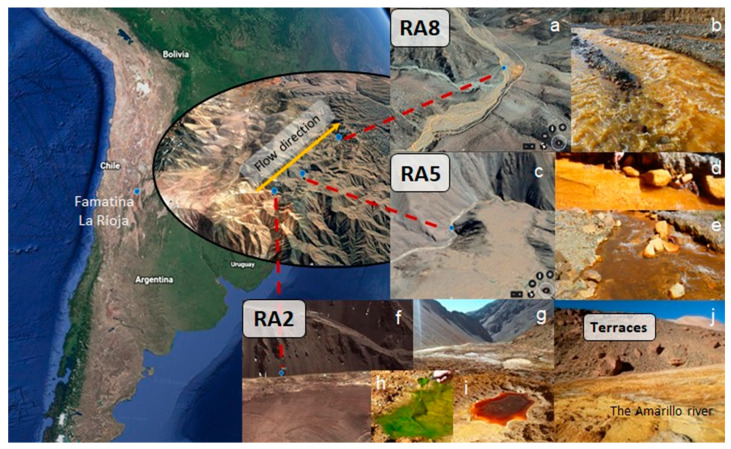
The right map shows the Amarillo River and the sampling point locations in La Rioja, Argentina. (**a**,**c**,**f**) Satellite images of the sampling sites RA8, RA5 and RA2 (including the sampling point of the Cueva de Perez formation), respectively. (**b**,**e**) Images of the Amarillo River yellow waters in sampling points RA8 and RA5, respectively. (**d**) Image of the ochreous-yellowish sediments present in the river basin at sampling point RA5. (**g**,**j**) Images of the Amarillo River and the terraces from the Cueva de Perez formation in the RA2 area. (**h**,**i**) Image of a green streamer and a red pool found in the RA2 area, respectively.

The Amarillo River and the Cueva de Perez terraces were sampled at three points each. Waters and bed sediments (river) or solid material (terraces) were aseptically collected. The river samples were named RA2, in the upper part of the river, near its origin on the mountain, and RA5 and RA8, in the middle part of the Amarillo River, after receiving neutral tributary courses. The name of the sampling points corresponds to the historical nomenclature used in the first geological studies of the river, and it does not imply numerical correlation. Figure 1 shows a map of the area and photographs of the sampling points, where the changes in the colours of the waters and sediments due to geomicrobiological processes can be observed.

The Amarillo River presents acidic waters throughout its course (pH around 3) in spite of receiving different alkaline tributary courses. Redox potential is also maintained between 575 and 716 mV. At the origin of the river, the temperature of the waters is close to 5 °C, and then it stabilises in approx. 10 °C and reaches 14 °C close to the RA8 sampling point. The river suffers an important dilution downstream, which is reflected, for instance, in the decrease in electrical conductivity and sulphate concentration (Table 1). Iron is the most relevant metal in the waters of the river, with a concentration of 1.2 g L^−1^ (mainly in the Fe(II) form) in RA2, decreasing to values close to 0.1 g L^−1^ in the RA8 sample (Table 1 and reference [7]). Table 1 shows the geolocation and the main physicochemical characteristics of the river waters at the sampling points. Water was filtered in situ with a pore size of 0.22 µm membrane. The values reported in a previous geological study [7] are presented between brackets for comparison. 

The basin of the Amarillo River is completed by discontinuous terraced deposits composed of clastic to matrix-supported conglomerates, with a sandy matrix and cemented with oxy/hydroxides/iron sulphates, called the Cueva de Pérez Formation [18]. Due to their stratigraphic position, these deposits were alternatively placed in the Early Pleistocene (approx. 2.58 million years ago) and Holocene (approx. 11.7 thousand years ago); therefore, these cemented terraces or ferricretes of the Cueva de Pérez Formation could be considered fossil analogues of the present-day river basin [19]. 

The samples from the Cueva de Perez terraces (ACP) were taken in the outcrop corresponding to the upper basin on the sides of the sampling area called RA2 of the Amarillo River. The names used for these samples correspond to Juarez [19], where the mineralogy of the area is described. ACP-28 refers to the samples collected in the vicinities of RA2, with 1, 3 or 4 referring to the height; sample ACP 281 is the topographically highest level and therefore the oldest.

### 2.2. Enrichment Cultures

The enrichment cultures (FeEC) were obtained via inoculation of a mixture of waters and sediments of the river samples in Mackintosh basal media [20] at pH 1.8 (to prevent abiotic Fe(II) oxidation and posterior hydrolysis and to favour the development of iron-oxidising microorganisms), with FeSO_4_·7H_2_O (0.16 M) without organic carbon (autotrophic, symbolised with A in the name of the culture) or with 2 g.·L^−1^ of yeast extract (heterotrophic, symbolised with H) and incubated at 4, 15 or 30 °C in agitation (150 rpm). The names of the enrichment cultures were constructed using the sample point, the temperature of cultivation and the carbon source; for instance, RA2-15A is the culture obtained from the river sample RA2, cultivated at 15 °C without yeast extract, while RA8-30H was obtained from the river sample RA8, cultivated at 30 °C and with 2 g.·L^−1^ yeast extract. Enrichment cultures were transferred successively to fresh media when Fe(II) was completely consumed, which could be between 5 and 20 days, mostly depending on the culture temperature. 

### 2.3. DNA Extraction and Sequencing

Genomic DNA was extracted from 1 g of each environmental sample (mixture of water and sediments for the river samples and solid material for the terraces samples) or 2 mL of each enrichment culture using Ultraclean Soil DNA Isolation Kit (MO BIO Laboratories, Carlsbad, CA, USA), following the manufacturer’s instructions. DNA samples were visualised on a 1% (*w*/*v*) agarose gel with ethidium bromide staining and quantified using a NanoDrop spectrophotometer (NanoDrop Technologies, Wilmington, NC, USA). Amplicon sequencing was performed via Molecular Research LP Mr DNA (Shallowater, TX, USA) in the Illumina MySeq platform, targeting the V3–V4 region of the prokaryotic 16S rRNA gene (341F-CCTACGGGNGGCWGCAG- and 785R-GACTACHVGGGTATCTAATCC-primers) for the six environmental samples and the four enrichment cultures and the Internal Transcribed Spacer region of the rRNA genes (ITS1F-CTTGGTCATTTAGAGGAAGTAA- and ITS2R-GCTGCGTTCTTCATCGATGC-primers) was chosen for fungi detection for the six environmental samples and the two heterotrophic cultures. 

### 2.4. Bioinformatic Analysis

Raw reads from the 16S rRNA gene and the ITS region were analysed using the open source bioinformatic pipeline QIIME2 v.2021.11. Inspection reads quality reported that the per-base quality was >35 across raw data. Determination of amplicon sequence variants (ASVs), filtering, dereplication, turning paired-end fastq files into merged, and removing of chimera sequences was carried out via Dada2 [21]. The feature tables generated were filtered out of ASVs that had a frequency of less than 0.1% of the mean sample depth. Bacterial ASVs matching “Chloroplast” and “Mitochondria” were removed from the data set. 

Bacterial taxonomic assignments were made using the pre-trained classifier silva-138-99-nb-classifier reference database. For the fungal identification, the classifier on the UNITE fungal ITS database was used (unite-ver-8-99-classifier-10.05.21).

The raw Illumina sequences are deposited in the NCBI database under the BioProject ID PRJNA994910.

The ASVs tables were processed using the phyloseq library [22] from RStudio (v. 2023.06.0 + 421). Richness and alpha diversity analysis were carried out using observed features (in this case, ASVs), and the Shannon index, Chao index and Simpson Index [23] were determined. Rarefaction curves were plotted using the vegan function rarecurve of the vegan package. All plots were carried out using ggplot2 (V 3.4.4).

Venn Diagrams: Exclusive and shared ASVs between samples were detected using a Venn diagram in R Studio using MicrobiotaProcess for calculations and ggVennDiagram for graphical representations. The final figure presented is a composition edited for better graphical comprehension of the information.

Network construction: A unique table was built with bacterial and fungal ASVs identified to the genus taxonomic level with relative abundance over >0.3%. Correlation Network Analysis (CNA) was performed using the Sparse Co-occurrence Network Investigation for Compositional Data (SCNIC) integrated into the q2-scnic plugin from the Qiime2 software toolkit. All the samples that had less than 500 features and all the features that had an average mean of less than 2 across all the samples were filtered out. Pairwise correlations between all ASVs in the dataset were calculated with the SPARCC algorithm (*p*-value 0.05, bootstrap 1000). The network was built from that correlation table using an R correlation-value cutoff of 0.5. The network constructed was visualised in Cytoscape (v. 3.9.1) software.

## 3. Results

### 3.1. High-Throughput Sequencing Metrics and Biodiversity Analysis

#### 3.1.1. Bacterial Diversity in the Amarillo River System and the Fe(II) Enrichment Cultures

The high-throughput sequencing of the V3–V4 region of the 16S rRNA gene of the ten samples resulted in a total of 89,440 clean sequences, which were grouped into 256 representative amplicon sequence variants (ASVs). 

**Figure 2:**
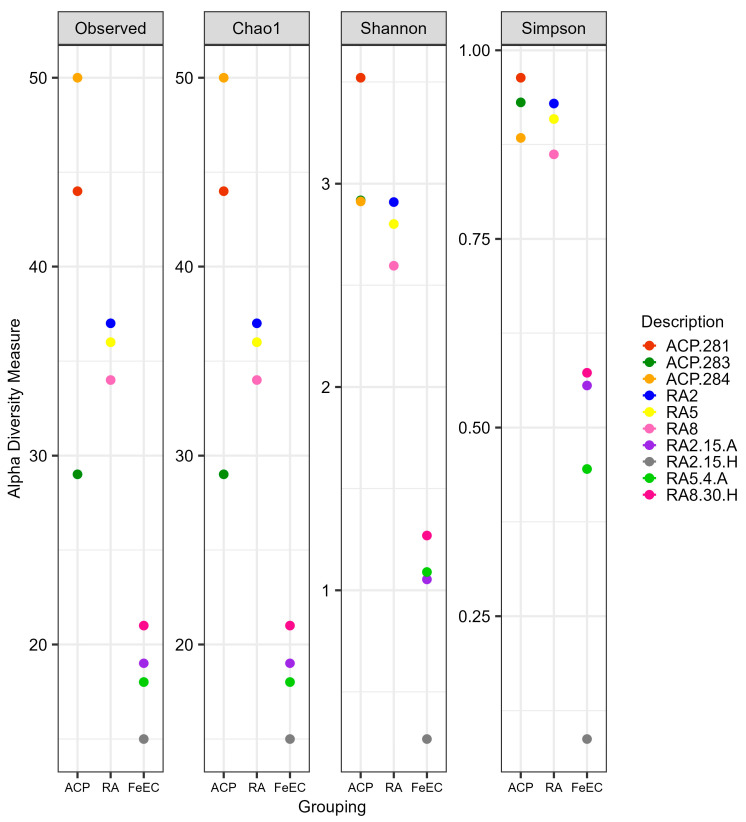
Alpha diversity indexes for bacteria ASVs. ACP: samples from the terraces of the Cueva de Perez formation. RA: Amarillo River. FeEC: Fe(II) enrichment cultures.

The alpha diversity at the ASV level of the ten samples was analysed using the classical indexes approach. Figure 2 shows the alpha diversity indexes Chao1 (abundance of species), Shannon (depending on richness and abundance, it quantifies the uncertainty in predicting the species identity of an individual that is taken at random from the dataset; the more homogeneous the sample, the more uncertainty) and Simpson (1-D, where D is a dominance index that measures the probability, between 1 and 0, that two individuals taken at random belong to the same species). As the Chao1 index coincides with the observed features for all the samples, it can be assumed that the bacterial diversity has been covered (rarefaction curves are presented in Supplementary Figure S1). 

The analysis of the diversity indexes shows that the environmental samples (river and terraces) were almost equally diverse and even. On the other hand, the enrichment cultures presented lower diversity indexes than the environmental samples. However, the Shannon and Sympson values were surprisingly high, indicating that the enrichment cultures presented more than one relevant ASV, except for culture RA2-15H. 

**Figure 3:**
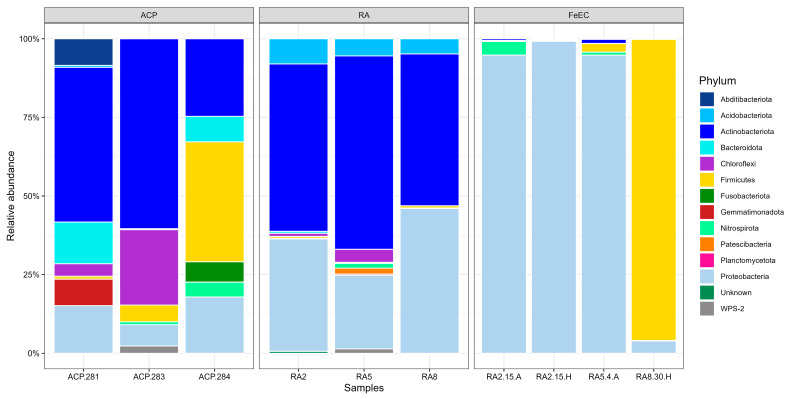
Bar plot of the relative abundance of bacterial phyla. “Unknown” collects all the sequences that could not be assigned to any phyla.

According to the taxonomic affiliation, all the sequences belonged to the Bacteria domain, and no Archaea were detected in any of the ten samples with the set of universal primers used, which might have biased the result against this domain. Regarding phylum (Figure 3), the environmental samples from the Amarillo River, RA2, RA5 and RA8, were dominated by Actinobacteriota (53.3%, 61.5% and 48.3%, respectively), followed by Proteobacteria (35.8%, 23.4% and 46.0%, respectively) and presented minor contributions of Acidobacteriota, Chloroflexi, Firmicutes and Nitrospirota (less than 5% in all samples except for Acidobacteriota in RA2 (8.0%) and in RA5 (5.4%)). In the samples from the terraces (ACP281, ACP283 and ACP284), Actinobacteria were still dominant (49.2%, 60.4% and 26.3%, respectively), and Proteobacteria were less represented (13.2%, 6.8% and 17.9%, respectively). The contribution of other phyla was not much relevant, except for 13.2% in ACP281 and 8.1% in ACP284 of Bacteroidota, 24.0% of Chloroflexi in ACP283 and 5.3% and 38.1% of Firmicutes in ACP283 and ACP284, respectively (Figure 3). On the other hand, the consortia obtained from the environmental samples collected in the Amarillo River, cultured at selective temperatures and in specific growth media to favour acidophilic iron-oxidising species, showed a high dominance (equal or over 95%) of only one phylum. The enrichment cultures RA2-15A, RA2-15H and RA5-4A were dominated by Proteobacteria and culture RA8-30H by Firmicutes, while the abundance of Actinobacteriota descended drastically (1.3% in RA5-4A and less than 0.6 in the others). 

**Figure 4:**
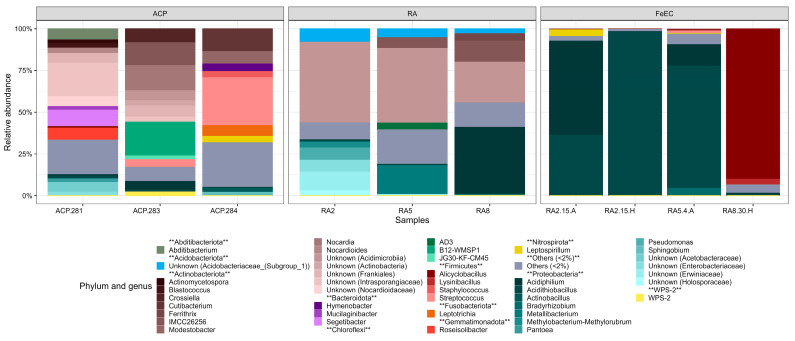
Bar plot of the most abundant bacterial genera listed by phylum. The sequences that could not be classified up to genus are listed as “Unknown”, with the deepest classification possible named between brackets. “Other” collects the sequences with abundances lower than 2%. ** indicates the phylum to which the genera listed below belong.

The ten samples presented higher diversity at the genus level than at phylum, but it was still possible to recognise the most prevalent taxa, especially in the samples from the Amarillo River and the enrichment cultures (Figure 4). In the river samples, RA2, RA5 and RA8, the most represented ASVs were associated with different unclassified or uncultured members of the Acidimicrobiia class in the Actinobacteria phylum. These ASVs were 99–100% similar to uncultured clones retrieved from different acid mine drainages where other species similar to the ones found in this study have also been detected ([24,25,26,27]) (Supplementary Table S1). Only a minority of the ASVs assigned to Acidimicrobiia were affiliated with known genera with cultivated representatives: *Ferritrix* (4.7% in RA8, 1.0% in RA2) and *Ferrimicrobium* (0.3% in RA5) (Supplementary Table S1). Within Proteobacteria, *Acidiphilium* (Alphaproteobacteria class) was the genus best represented, especially in RA8, where it comprised 40.7% of the sequences (1.0% in RA2 and 1.7% in RA5). The next most abundant genus within Proteobacteria was *Metallibacterium* (Gammaproteobacteria class), only present in RA5 with a 17.5% of relative abundance. In sample RA2, the most abundant taxa within the Proteobacteria class were related to unclassified Enterobacteriales and *Pseudomonas*. The class Acidobacteriae (Acidobacteriota phylum) was significantly represented in the river (RA2 8.0%, RA5 5.4% and RA8 4.8% relative abundance) mostly by uncultured species comprised in only one ASV 100% similar to an uncultured clone retrieved from an acid mine drainage exposed to extremely contrasting continental seasonal temperature patterns [21] (Supplementary Table S1).

The enrichment cultures, specifically designed to favour iron-oxidising species, were less diverse and dominated by one or two genera. These dominant genera could be composed of different ASVs, which would explain the values of the Shannon and Sympson indexes (Figure 2). The most extreme case was culture RA2-15H, which showed a 98.7% relative abundance of *Acidithiobacillus* and less than 0.5% of other acidophiles like *Alicyclobacillus*, *Acidiphilium* and uncultured members of the Acidimicrobiia class (Supplementary Table S1). Particularly, this culture presented the lowest Shannon and Sympson values (Figure 2), as it is composed mostly of only one ASV (95.5% relative abundance, Supplementary Table S1). Culture RA2-15A had two main genera contributing to its biodiversity: *Acidithiobacillus* (36.6% relative abundance) and *Acidiphilium* (57.9%), complemented by 4.3% of *Leptospirillum*. Culture RA5-4A was also dominated by *Acidithiobacillus* (75.9%), complemented by 12.7% of *Acidiphilium* and showed a minor presence of *Alicyclobacillus* (0.8%) and *Leptospirillum* (0.9%). Lastly, culture RA8-30H was dominated by *Alicyclobacillus* (91.0%) and complemented by *Lysinibacillus* (3.6%), *Acidithiobacillus* (0.7%), *Acidiphilium* (0.8%) and *Leptospirillum* (0.1%). 

These acidophilic, lithotrophic sulphur and/or iron-oxidising genera that dominated the Fe(II) enrichment cultures and are typically related to the iron cycle in acidic environments were less than 1% represented in the Amarillo River; for instance, *Acidithiobacillus* was detected with a relative abundance between 0.7 and 0.8% in RA2, RA5 and RA8, while *Leptospirillum* was detected with a relative abundance of 1.5%, but only in RA5. 

In the terraces, the most represented genera were *Streptococcus* (29.5% in ACP-284 and 4.6% in ACP-283), *Cutibacterium* (13.7% in ACP-284 and 0.8% in ACP-283), genus B12-WMSP1 (Cloroflexi, 21.3% in ACP-283), *Modestobacter* (7.7% ACP-284) and unclassified members of the Intrasporangiaceae family (Actinobacteriota phylum, 20.1% ACP-281, 3.1% ACP-283 and 0.5% in ACP-284). The acidophilic iron-oxidising genera prevalent in the enrichment cultures were also detected in the terraces, with relative abundances similar to those found in the river (Figure 4, Supplementary Table S1). The different species of the genus *Acidithiobacillus* comprised 1.7%, 1.1% and 0.7% of abundances in ACP-281, ACP-283 and ACP-284, respectively, while *Acidiphilium* was detected with 0.8%, 5.1% and 0.4% of relative abundance in the same samples and *Alicyclobacillus*’s percentual abundances were 0.9, 0.4 and 0.7. *Leptospirillum* was found with a 0.9% abundance in ACP-283 and a 4.7% in ACP-284. 

#### 3.1.2. Fungal Diversity in the Amarillo River System and the Fe(II) Enrichment Cultures

In the case of Fungi, the ITS1 region was analysed via metagenomic sequencing in the three samples from the Amarillo River, the three samples from the terraces and the heterotrophic cultures RA2-15H and RA8-30H. The analysis of the eight samples resulted in a total of 482,631 clean sequences, which were grouped into 206 different ASVs. 

**Figure 5:**
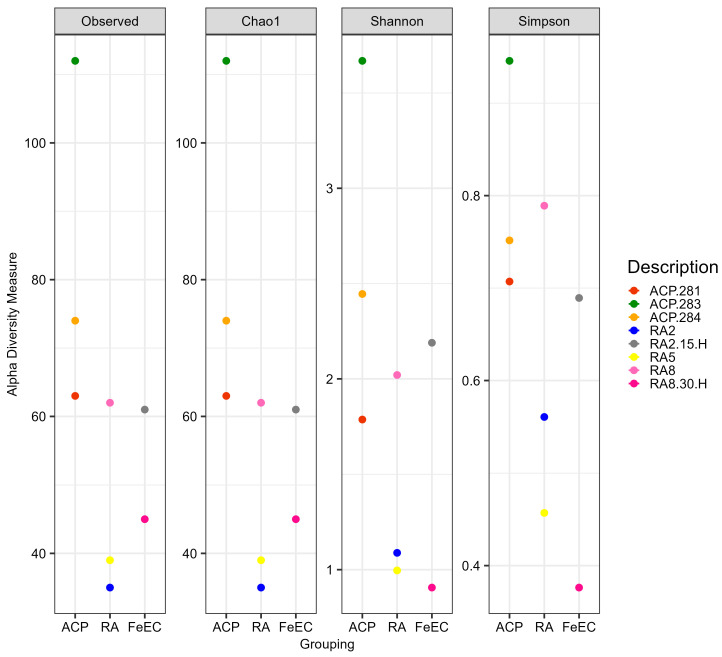
Fungal alpha diversity indexes. ACP: samples from the terraces of the Cueva de Perez formation. RA: Amarillo River. FeEC: Fe(II) enrichment cultures.

The diversity indexes presented in Figure 5 show that all the samples, including the cultures, had quite diverse fungal communities, particularly in the terraces. As was previously stated for bacteria, the Chao1 index coincides with the observed features in all the samples; therefore, fungal diversity has been covered (confirmed via the rarefactions curves presented in Supplementary Figure S1).

At the phylum level, only two phyla described almost all the fungal diversity: Ascomycota, which comprises approx. 75% of abundance in the samples from the terraces and 99% in the heterotrophic cultures and Basidiomycota. Two other unidentified phyla represented 8.7% of abundance in sample ACP-281 and 2.7% in ACP-283, and Mortierellomycota, only represented 0.3% of abundance in sample ACP-288.

**Figure 6:**
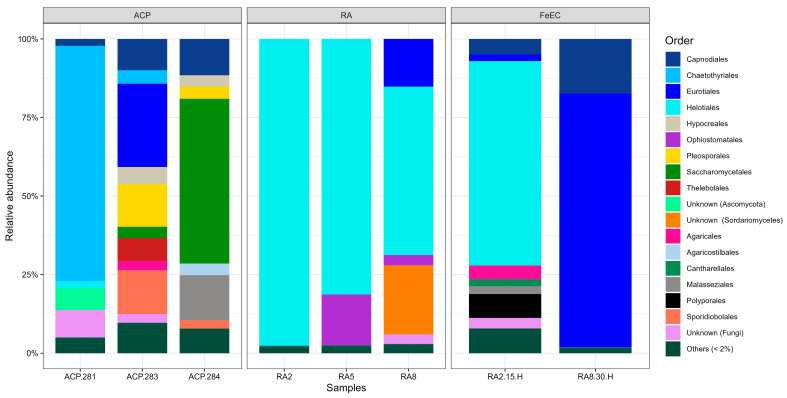
Bar plot of the relative abundance of fungal orders. “Unknown” collects all the sequences that could not be assigned to any order. “Other” collects all the orders that presented abundances lower than 2%.

**Figure 7:**
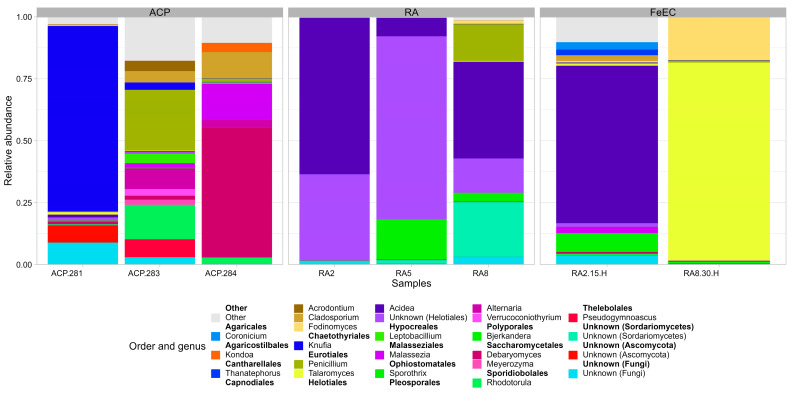
Bar plot of the most abundant fungal genera listed by order. The sequences that could not be classified up to genus are listed as “Unknown”, with the deepest classification possible named between brackets. “Other” collects the sequences with abundances lower than 2%.

At the order level (Figure 6), the samples from the Amarillo River were clearly dominated by Helotiales (RA2 97.6%, RA5 81.4% and RA8 53.6%), while in the samples from the terraces, this taxon had low abundance (<2%). The samples from the terraces were more diverse at the order level than the ones from the river, and the dominant order was different in each one, consistent with the fact that the different heights represent different geological times: ACP-281 was dominated by Chaetothyriale order (74.0%); ACP-283 by Eurotiales (26.5%); and ACP-284 by Saccharomycetales (52.5%) (Figure 6). The relative abundances of fungal genera are presented in Figure 7. The most abundant species in the river samples were related to *Acidea extrema* (Helotiales order) (RA2 63.1%, RA5 7.43.0% and RA8 39.1%, relative abundance), a relatively novel genus of hyaline fungus isolated from extremely acidic soils an able to grow at a range of pH from 1 to 7 at 24 °C [15]. This was also the most abundant species in the heterotrophic culture at 15 °C (RA2-15H 70.9% abundance). However, it was almost undetected in the heterotrophic culture at 30 °C (RA8-30H 0.4%), probably because of the temperature above its optimum. Within the order Helotiales, another group not further classified (Unknown Helotiales in Figure 7) was detected with high abundance in the river (RA2 34.4%, RA5 73.9% and RA8 13.8%). Other relevant species identified in the river were related to the genus *Sporothrix* (order Ophiostomatales; RA5 16.1%, RA8 3.3%), commonly found in soils and plants, to an uncultured fungus only classified up to the class *Sordariomycetes* (RA5 1.4%, RA8 22.0%) whose closest relatives were also retrieved from high altitude environments ([28,29]), and to the ubiquitous genus *Penicillium* (RA8 14.8%).

In the terraces, one of the most abundant fungal species was associated with the genus *Knufia* (Chaetothyriales order) (ACP-281 74.9%), a rock-inhabiting black fungus that produces high amounts of extracellular polymeric substances (EPS) as an adaptation mechanism to survive temperature fluctuation, desiccation and prolonged exposure to UV radiation [30], all stressful characteristics of the Cueva de Perez terraces. Other genera relatively abundant in the terraces were *Debaryomyces* (Saccharomycetales order) with an abundance of 52.5% in ACP 284; *Penicillium* (Eurotiales order); and *Rhodotorula* (Sporidiobolales order), an EPS and carotenoids producing environmental yeast ([31,32]) with 24.6% and 13.9% abundance, respectively, in sample ACP-283. Other genera detected, with at least 7% abundance in one terrace sample, were *Alternaria* (Pleosporales order) and *Cladosporium* (Capnodiales order), both of which are cosmopolitan species; *Malassezia* (Malasseziales order), known for causing skin disease; and *Pseudogymnoascus* (Thelebolales order), a diverse group inhabiting a wide range of cold environments [33].

Regarding the enrichment cultures, as mentioned, culture RA2-15H was dominated by *Acidea* (63.5%), followed by *Bjerkandera* (7.6%), a genus of Basidomycota known for their dye degrading capacity [34]. On the other hand, culture RA8-30H was dominated by (80.0%) *Talaromyces*, followed by *Fodinomyces* (17.3%), a genus of Ascomycota detected for the first time in a uranium mine [35].

Finally, the taxonomic classification of a significant proportion of the fungal ASVs found could not be fully elucidated, even using the latest versions of the most modern databases (referred to as Unknown in Figure 6 and Figure 7). 

## 4. Discussion 

### 4.1. Species Involved in Iron Metabolism

The enrichment cultures carried out with ferrous iron as the main electron source at 4 and 15 °C with and without carbon source allowed for the highlighting of the members of the microbial community that are probably most actively involved in iron metabolism in the different environmental and climatologic conditions of the Amarillo River along the year. 

*Acidithiobacillus ferrivorans* is present in the river, as well as the terraces, and seems to be the main iron oxidiser at the temperature range of the river as it was the most abundant species in the enrichment cultures at 4 and 15 °C, with or without yeast extract (Figure 4 and Supplementary Table S1). *A. ferrivorans* is an obligate autotroph able to use ferrous iron and reduced sulphur compounds as electron donors. It is mesophilic but able to grow at 4 °C [36], as proven by its high abundance in culture RA5-4A (74.9%) and the remarkable performance of this culture in ferrous iron oxidation and ferric iron precipitation [9]. The genomic analysis of *A. ferrivorans* showed certain traits that might give this strain certain advantages compared to other *Acidithiobacillus* and could help to explain its prevalence in extreme environments like the Amarillo River; for instance, it has multiple iron oxidation systems represented by the genes rusA (present in all *Acidithiobacillus*), rusB1, rusB2 (isoforms of rusticyanin) and iron periplasmic high potential iron–sulphur protein (HiPIP), an electron acceptor involved in ferrous iron oxidation in bacteria and the presence of genes associated with the oxidation of reduced sulphur compounds under denitrifying conditions ([37,38,39]). Also, some strains of *A. ferrivorans* produce large amounts of EPS that contribute to the formation of biofilms and streamers [39], like the ones that can be seen in the Amarillo River (Figure 1h).

*Acidiphilium* is another relevant species in the microbial community of the river as it was detected in all six environmental samples, with a surprisingly high relative abundance in RA8 (40.6%) and in the four enrichment cultures, being dominant in culture RA2-15A. *Acidiphilium* are acidophilic, mesophilic, chemoorganotrophic bacteria able to oxidise sulphur compounds but not ferrous iron, although its addition stimulates heterotrophic growth. The genus *Acidiphilium* shows an interesting metabolic flexibility that probably allows its development in different extreme environments and in conditions distant from its growth optimum. There are species capable of chemolithoautotrophic growth, ferric iron reduction in anaerobic conditions and arsenite oxidation, but probably the most surprising treat is the production of photopigments with zinc-chelated bacteriochlorophyll (Zn-BChl) that are much more stable in acidic conditions than other photopigments ([40,41]). Even though there is no evidence of actual photosynthetic growth in *Acidiphilium*, considering that they develop mostly in acidic, low-carbon environments, such ability may be an adaptation that would help explain its survival in hostile conditions ([40,41]). *Acidiphilium* participation in the biogeochemical iron cycle is associated with the reduction in ferric iron in oxic–anoxic interfaces, regenerating ferrous iron that can be used by iron-oxidising species. Precisely, this could be the part it is playing in the Amarillo River, as it is highly abundant downstream (sample RA8) where most of the ferrous iron found upstream has already been oxidised (Table 1). 

Members of the Acidimicrobiia class seem to be an important part of the microbial community of the Amarillo River; however, little can be said about their participation in its geomicrobiology as most of them were associated with uncultured species, except for *Ferrithrix*, which was detected in RA5 and RA8 (1.00 and 4.7% relative abundance, respectively), and *Ferrimicrobium*, which was detected only in RA5 (0.3% relative abundance). At present, the class Acidimicrobiia only contains two families, Acidimicrobiaceae and Iamiaceae (with no genomes sequenced), and very few cultivated species, all of them isolated from extremely acid environments and most of them able to oxidise ferrous iron [42]. *Ferrithrix* are obligate heterotrophs, acidophilic (optimum pH 1.8) and moderate thermophilic (optimum growth temperature 43 °C) bacteria that are able to oxidise and reduce iron [43]. *Ferrimicrobium* have very similar characteristics, although they are mesophilic (optimum growth temperature 35 °C; maximum 37 °C), and they are able to oxidise pyrite in addition to ferrous iron [43]. The Acidimicrobiia of the Amarillo River failed to grow in the acidic enrichment cultures, showing once more the difficulty they present for cultivation in spite of their high abundance. The sequences associated with Acidimicrobiia were detected in the enrichment cultures with relative abundances lower than 0.5 using high-throughput sequencing (Supplementary Table S1). Despite this, their presence in the river, especially *Ferrithrix* and *Ferromicrobium*, might indicate the flexibility and potential of iron oxidation in the system at a variety of environmental conditions. All the ASVs affiliated with Acidimicrobiia were very closely related to uncultured clones retrieved from diverse acidic, high metal concentration environments like the Iberian Pyritic Belt and Río Tinto, Río Agrio (a natural acidic river in Neuquén, Argentina), different acid mine drainages, acidic pit lakes and naturally acidic springs ([3,24,25,26,27]). The global occurrence of different members of the Acidimicrobiia class evidently points to their importance in acidic microbial communities. Consequently, it is still a challenge for science to answer at least two questions; why they are so reluctant to cultivation and isolation, and, in a second step, which is their geomicrobiological role in acidic environments. Places like the Amarillo River, with a high abundance of Acidimicrobiia, could be interesting for deeper metagenomics studies to attempt to answer these questions. 

In order to test the flexibility of the system regarding iron oxidation, we made an enrichment culture with ferrous iron as the main electron source and yeast extract as the carbon source at 30 °C, a temperature rarely found in the Amarillo River. The microbial consortium selected (R8-30H), which was able to oxidise ferrous iron and to form ferric iron precipitates at a rate much higher than the other enrichment cultures at 15 or 4 °C [9], was dominated by *Alicyclobacillus* (91.0% relative abundance; Figure 4), a moderately thermophilic, acidophilic, strictly aerobic and endospore-forming bacterium that can grow mixotrophically using ferrous iron or reduced sulphur compounds [44]. *Alicyclobacillus* has been found in all samples along the Amarillo River and the terraces as well as in the cultures at 4 and 15 °C. Its survival in highly acidic conditions or others distant from its optimum for growth has been associated with the high abundance of ω-cyclohexane fatty acids in its membranes and its sporulation capacity [44]. Although different *Alicyclobacillus* species are frequently detected in molecular ecology surveys in highly acidic extreme environments, natural or anthropogenically generated ([3,24]), they have rarely been isolated and consequently studied, especially at the genomic level. In spite of being mainly heterotrophs, in many cases, *Alicyclobacillus* are the predominant iron-oxidising species, even though they generally oxidise at lower rates than other mesophilic or moderate thermophilic iron oxidisers ([45,46]). *Al. tolerans* AMF was the main iron oxidiser in the bioleaching of a violarite–pentlandite–chalcopyrite concentrate, highlighting the role of mixotrophs in this kind of process. As in culture RA8-30H, the *Alicyclobacillus* strain was accompanied, although in lesser abundances, by *Acidithiobacillus* species and other iron and/or sulphur oxidisers [45]. Another study showed that *Al. ferrooxydans* and *Al. ferripilum* were the dominant heterotrophs capable of iron oxidation in a simultaneous sludge digestion and metal leaching process [47]. Demir and co-workers [25] studied iron oxidation in a ceramic membrane bioreactor at room temperature using acidophilic bacteria isolated from an acid mine drainage in Fe(II) and tryptone soya broth medium, and they found that iron oxidation reached almost 100% when the abundance of *Alicyclobacillus* increased from 40% to 78% [25]. Even when *Alicyclobacillus* might not be the best iron oxidiser, heterotrophic metabolism generates higher cell densities much faster than lithoautotropic metabolisms (particularly the oxidation of ferrous iron provides very little energy and reductive power), allowing for iron-oxidising chemolithotrophic species, such as *Alicyclobacillus* and others, to colonise and take over iron oxidation [48]. In this context, the Amarillo River, and particularly culture R8-30H, could serve as an interesting starting point to further study environmental iron-oxidising *Alicyclobacillus*. 

Regarding fungi, none of the known and characterised species detected in any of the samples have, to today’s knowledge, direct participated in the environmental iron cycle, meaning they are not capable of oxidising iron by themselves, even though fungi are important players in the nutrients cycle in acidic extreme environments as main decomposers of organic matter as well as in biomineralisation ([13,49,50,51,52]). However, considering the significant proportion of unknown, unclassified fungal ASVs found, the actual role of fungi in the iron cycle may be different from what we can infer from current knowledge. 

### 4.2. Fungi in the Amarillo River

The high number of fungal ASVs in the river and the terraces suggest an important ecological role of fungi in the present and past of the Amarillo River.

Fungal species are known to be more tolerant to harsh physicochemical conditions (low pH, low water activity, high metal concentrations) than bacteria. Rousk and co-workers [53] showed in their study of soils with pH ranges from 4 to 8 collected from different environments in North and South America that bacterial relative abundance and diversity were highly affected by pH, while fungal abundance and diversity in the same samples were almost unaltered. In more acidic soils (pH < 3), mycodiversity maintains high but tends to be more specific, with higher abundances of acidophilic species ([15,54]), a result also found in the Amarillo River, dominated by the acidophilic genus *Acidea*. On the other hand, the terraces showed a more diverse and even community, with the exception of sample ACP281 dominated by *Knufia*, a rock-inhabiting microcolonial fungi genus well adapted to survive in low water availability and high UV radiation environments, as are the terraces of the Cueva de Perez [30]. 

Most of the known and classified fungal species found in this work have been previously reported in acidic, cold environments or with high concentrations of heavy metals. All the reported species of *Acidea* were capable of growth at pH 3 or lower and some have been isolated from Antarctic environments. It has been reported that some members of the genus *Penicillium* are capable of growing in acidic waters with high concentrations of heavy metals ([55,56,57]). In addition, some psychrophilic *Penicillium* species have also been described ([58,59,60]. Yeast from the genus *Rhodotorula* has been reported in the Tinto River and Antarctic soil ([56,61]) together with species of *Debaryomyces* [61].

The fungal species that live in the Amarillo River, as well as other similar extreme environments, also need to deal with the high concentration of many heavy metals, including iron. There are multiple studies that demonstrate fungal resistance to heavy metals, even in concentrations higher than those detected in the Amarillo River; in general, the cell wall of fungi shows excellent metal-binding properties, allowing them to adapt and grow in these extreme conditions (reviewed in [51]).

Finally, the high prevalence of incompletely classified sequences observed in this study serves as a compelling testament to the existing limitations in fungal taxonomic databases. Furthermore, it emphasises the pressing need for expanded research efforts to investigate the fungal diversity in extreme environments, revealing an extensive reservoir of unexplored fungal taxa that await thorough exploration, comprehensive description, high-throughput sequencing, and accurate classification.

### 4.3. Common Species and Interactions

**Figure 8:**
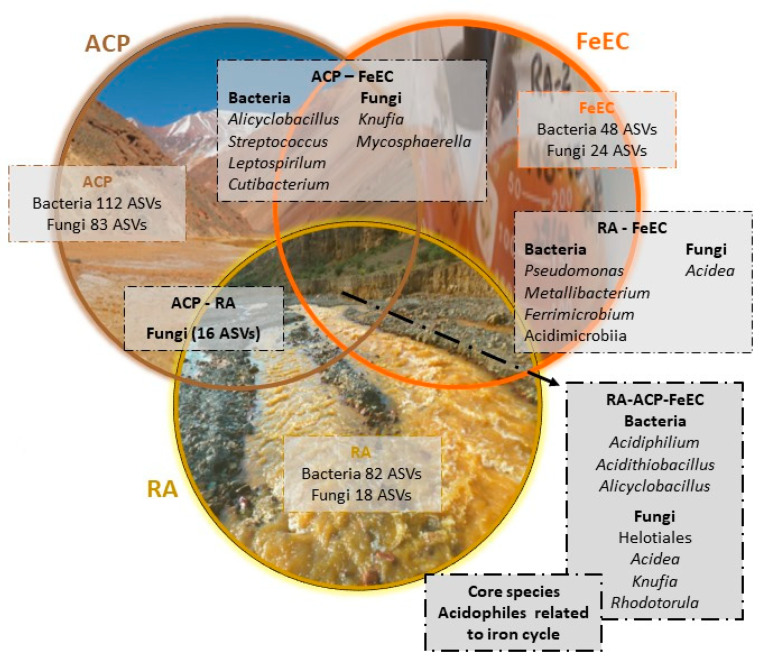
Veen diagram showing common bacterial and fungal species among the terraces (ACP), Amarillo River (RA) and the Fe(II) enrichment cultures (FeEC). The analysis was then conducted at the ASV levels separately for bacteria and fungi. Taxon names correspond to the taxonomic affiliation of the ASV according to the UNITE database. All bacterial common taxa are listed. In the case of fungi, only the most relevant taxa were listed. For a complete list of fungal common taxa see Supplementary File S1.

To better understand the microbial community that develops in the Amarillo River and its fossil analogues, the terraces of the Cueva de Perez formation, with special emphasis on the microorganisms that might be involved in iron metabolism, we performed different analyses. The Venn diagram of Figure 8 shows the common taxa among the three groups of samples studied. This graphic representation allowed us to recognise the core species of the system and reinforced the importance of the acidophilic and iron-oxidising species. The common species among the tree systems (Amarillo River (RA), terraces (ACP) and iron enrichment cultures (FeEC)) were related to the genera *Acidithiobacillus*, *Acidiphilium*, *Acidea*, *Knufia*, *Rhodotorula* and unclassified species from the Helotiales order. Probably, *Alicyclobacillus* should be added to this core, as it was detected in the river, terraces and enrichment cultures in different ASVs that match the exact same sequences with 100% identity, but they are not all in all the samples (Supplementary Table S1). Even more, the fact that there are no other common bacteria among the river and terraces (RA-ACP box in Figure 8) besides the ones that are also common to the iron enrichment cultures highlights the present and past key role of the species involved in the iron cycle, in spite of their low abundances in the environmental samples.

**Figure 9:**
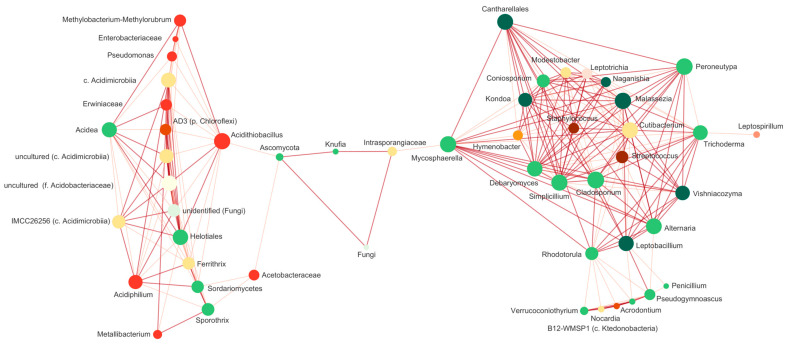
Bacterial and fungal co-occurrence network based on ASVs with relative abundances > 0.3%. Co-occurrence relations are represented by the edge lines. Only positive correlations with r > 0.6 are shown (based on the Spearman correlation test). Strong co-occurrence relations (r > 0.75) are represented by thicker edge lines. Nodes correspond to bacterial (shades of orange to red) or fungal (shades of green) ASVs. The size of the nodes (degree) represents the interactions of that node with the others.

On the other hand, the network analysis provided valuable insights into the interactions between bacteria and fungi in the Amarillo River and its terraces (Figure 9) without the bias induced by the iron enrichment cultures. The resulting network showed a great number of connections, with 51 nodes, 633 edges, an average number of neighbours of 24.82 and a network diameter of 3, which indicates a rather compact network where all the nodes are communicated [62]. There are two distinct clusters; one with most of the fungal species and various heterotrophic and mesophilic bacterial species, some associated with the human normal or pathogenic microbiome (*Staphylococcus*, *Streptococcus*, *Nocardia, Cutibacterium* and *Leptotrichia*) and other oligotrophic species detected in extreme, low-carbon, not acidic environments, such as *Hymenobacter* (uranium mine [63]) and *Modestobacter* (hyper-arid environments [64]), an uncultured clone from the genus B12-WMSP1 (Atacama desert [65]). The other cluster bares the species associated with acidophilic environments and the iron cycle: *Acidithiobacillus*, *Acidiphilium*, *Ferrithrix* and the other uncultured Acidimicrobiia, *Metallibacterium*, a moderate acidophile able to produce ammonium, which raise pHs in its vicinity [66], and the fungal specie *Acidea* and the members of the Helotiales that were related to it. A notable exception is an obligate acidophile, iron oxidiser *Leptospirilum*, located in the cluster with the non-acidophilic species in the opposite extreme to the other cluster and only interacting with a few species. Considering that *Leptospirilum ferrooxidans* is physiologically similar to *Acidithiobacillus ferrivorans* (the species that have 100% similitude to the ASVs detected (Supplementary Table S1), and it has been detected in the river as well as the terraces, its location in the network could not be completely understood. The nodes in both clusters were highly connected; however, the interaction among the clusters was mediated by only a few species, apparently very important for the community ensemble. Unfortunately, for most of them, it was not possible to have a complete taxonomic classification, except for *Knufia*. The presence of *Knufia*, a black microcolonial fungus, in the river samples is a noteworthy observation in our study. This genus, identified across the Rio Amarillo system and the iron enrichment cultures, plays a crucial role in connecting distinct clusters within the microbial communities. The resilience of this genus to harsh conditions, including desiccation, high UV radiation, and abrupt temperature changes, has been well-documented [67]. This adaptability likely contributed to its survival in both the extreme environment of the terraces of Cueva de Perez and in the acidic waters of Río Amarillo. The versatility of *Knufia* is evident in its ability to utilise diverse carbon sources and produce rock-corrosive extracellular polymeric substances [68]. This adaptability positions them as keystone species, probably essential for the survival of other community members, for instance, providing specific nutrients derived from rocks in the challenging conditions of the Río Amarillo system, characterised by limited available resources, and it helps to comprehend its connecting position between the acidophilic and non-acidophilic clusters. 

The clear topology of the network highlights the importance of the interactions among acidophiles and the species involved in the iron cycle in this complex extreme environment, which probably determine their survival and their role in the biogeochemistry of the landscape. 

## 5. Final Comments and Conclusions

The biodiversity and geomicrobiology of the Río Amarillo system are yet to be fully understood. Pioneering previous work brought to light the presence of bacteria and acidophilic fungi in consortia established from samples taken from the Amarillo river, confirming that their presence accelerates Fe(II) oxidation and consequently increases the formation of Fe(III) bearing minerals such as jarosite, present in the sediments accumulated in the river bed which gives it its characteristic yellow colour [9]. The present work not only confirms the presence of all the acidophilic iron cycle-related microorganisms previously reported using a much more robust sequencing technique but also unveils the presence of the species participating in the biogeochemical conceptual model in the environmental samples taken both in the river and the terraces, confirming the importance of their activity in influencing the geomicrobiology of the environment both now and in the past. The application of high-throughput sequencing techniques, coupled with advanced bioinformatic and biostatistical tools, not only enhances the resolution of the geomicrobiological model of the iron cycle but also brings to light previously overlooked, yet significant contributors such as *Acidiphilium*, *Alicyclobacillus* and even fungal species like members of the Heliotiales and the genus *Knufia*, even though their roles are not yet clear.

Finally, while we acknowledge that the limited number of samples analysed may impact the diversity results, and further deeper work will be needed to decipher the relations between the members of the microbial community and the environmental conditions and biogeological processes, it is essential to address this within the unique context of extreme environments. In contrast to more diverse ecosystems, extreme environments often exhibit a naturally lower microbial diversity; therefore, in such settings, the depth of sampling, while important, may not be as critical as in ecosystems with higher biodiversity. The observed consistency in microbial species across the Rio Amarillo system, as well as the Fe(II) enrichment cultures, and the plateau in rarefaction curves indicate that, within the constraints of our study, we captured a substantial portion of the microbial diversity, particularly that relevant to the biogeochemical iron cycle.

In conclusion, this study provides valuable insights into the complex relationships between bacterial and fungal communities in the formation of the iconic yellow landscape in the Amarillo River. By employing Venn diagrams and co-occurrence networks, we have shed light on their intricate interconnection. *Acidithiobacillus ferrivorans* has emerged as the main iron oxidiser, while *Acidiphilium*, despite its inability to oxidise ferrous iron, exhibited a metabolic flexibility that might play a significant role in the reduction in ferric iron, regenerating ferrous iron to be utilised by other iron-oxidising species. The dominance of *Alicyclobacillus* in the enrichment culture at 30 °C and its presence throughout the Río Amarillo system also point to its adaptability regarding iron oxidation to varying environmental conditions. On the other hand, this study represents the first comprehensive exploration of fungal diversity in the Amarillo River system. Our findings highlight the presence of acidophilic fungi from the order *Heliotiales* and the microcolonial black fungus *Knufia*. The detection of *Knufia*, known for its resilience in challenging conditions, enriches our insights into microbial dynamics in this extreme environment while leaving the question of what its role is in the interaction between the acidophilic and non-acidophilic members of the community. The discovery of unidentified Acidimicrobiia species, a repetitive component of microbial diversity in acidic environments, encourages additional research into their roles in extreme acidity. The presence of these microorganisms in the river, as well as in the prehistoric terraces, suggests their long-standing importance in the iron cycle. Collectively, these findings reinforce the importance of the microbial community as fundamental geological agents in the Amarillo River, where their collaborative work in the precipitation of iron minerals paints the landscape yellow.

## Figures and Tables

**Table 1:** Geolocalisation of the river points and main physicochemical characteristics of water samples. b.d.l. below detection limit (0.03 mg·L^−1^). * Values published by Lecomte and co-workers added for comparison [7].

Sampling Point Name	RA2	RA5	RA8
Latitude (S)	28°59′51.6″	28°58′34.5″	28°55′36.7″
Longitude (W)	67°44′21.4″	67°42′53.9″	67°40′34.0″
Height (m.a.s.l.)	3869	3419	2838
Downstream distance (Km)	4.89	8.83	17.09
pH	2.77 (* 3.00)	* 2.92	2.94 (* 3.33)
Eh (mV)	575 (* 667)	* 664	716 (* 634)
Conductivity (mS·cm^−1^)	6.7 (* 5.78)	* 4.15	1.63 (* 2.09)
Al (mg·L^−1^)	690 (* 542)	* 419	102 (* 185)
As (mg·L^−1^)	1.05 (* 0.51)	* 0.12	b.d.l.
K (mg·L^−1^)	58.8 (* 50)	* 13.6	7.4 (* 6.9)
Mg (mg·L^−1^)	76.6 (* 94.2)	* 73.9	29 (* 44)
Mn (mg·L^−1^)	47.7 (* 65.2)	* 42.3	11.4 (* 19.6)
Si (mg·L^−1^)	36 (* 39.7)	* 36.7	14.6 (* 21.2)
Ca (mg·L^−1^)	51.8 (* 41.6)	* 38.5	26.4 (* 33.2)
Cu (mg·L^−1^)	63.3 (* 39.9)	* 32.5	7.89 (* 13.6)
Fe_TOT_ (mg·L^−1^)	1430 (* 1220)	* 761	108 (* 196)
Zn (mg·L^−1^)	40.3 (* 50.9)	* 31.8	7.46 (* 14.2)
SO_4_^−2^ (mg·L^−1^)	2340 (* 5241)	* 4013	363 (* 1845)

## Data Availability

The raw Illumina sequences are deposited in the NCBI database under the BioProject ID PRJNA994910.

## Supplementary Materials

The following supporting information can be downloaded at https://www.mdpi.com/article/10.3390/microorganisms12020235/s1. Supplementary Figure S1. Rarefaction curves Bacteria and Fungi; Supplementary File S1. Venn diagram fungal species; Supplementary Table S1. Description of bacterial ASVs. References [24,25,26,27] are cited in the supplementary materials.

## References

[1] Hedrich S., Schipper A. (2021). Distribution of acidophilic microorganisms in natural and man-made acidic environments. Curr. Issues Mol. Biol..

[2] Amils R. (2016). Lessons learned from thirty years of geomicrobiological studies of Río Tinto. Res. Microbiol..

[3] Lopez Bedogni G., Massello F., Giaveno A., Donati E., Urbieta M. (2019). A deeper look into the biodiversity of the extremely acidic Copahue volcano- Río Agrio system in Neuquén, Argentina. Microorganisms.

[4] Kelly L., Rivett D., Pakostova E., Creer S., Cotterell T., Johnson D. (2023). Mineralogy affects prokaryotic community composition in an acidic metal mine. Microbiol. Res..

[5] Kuang J., Huang L., Chen L., Hua Z., Li S., Hu M., Li J., Shu W. (2013). Contemporary environmental variation determines microbial diversity patterns in acid mine drainage. ISME J..

[6] Brodtkorb M., Schalamuk B., Zappettini E.O. (1999). Yacimientos de cobre y oro de la Sierra de Famatina, La Rioja. Recursos Minerales de la República Argentina.

[7] Lecomte K., Maza S., Collo G., Sarmiento A., Depetris P. (2017). Geochemical behavior of an acid drainage system: The case of the Amarillo River, Famatina (La Rioja, Argentina). Environ. Sci. Pollut. Res..

[8] Maza S., Collo G., Astina R., Nieto F., Nieto J.M. (2014). Holocene ochreous lacustrine sediments within the Famatina Belt, NW Argentina: A natural case for fossil damming of an acid drainage system. J. S. Am. Earth Sci..

[9] Bernardelli C., Maza S., Lecomte K., Collo G., Astini R., Donati E. (2021). Acidophilic microorganisms enhancing geochemical dynamics in an acidic drainage system, Amarillo River in La Rioja, Argentina. Chemosphere.

[10] Johnson B., Kanao T., Hedrich S. (2012). Redox transformations of iron at extremely low pH: Fundamental and applied aspects. Front. Microbiol..

[11] Tiquia-Arashiro S., Grube M. (2019). Fungi in Extreme Environments: Ecological Role and Biotechnological Significance.

[12] Coleine C., Stajich J., Selbmann L. (2022). Fungi are key players in extreme ecosystems. Trends Ecol. Evol..

[13] Aguilera A., Olsson S., Puerte-Sánchez F., Quatrini R., Johnson D.B. (2016). Physiological and Phylogenetic Diversity of Acidophilic Eukaryotes. Acidophiles: Life in Extremely Acidic Environment.

[14] Das B., Roy A., Koschorreck M., Mandal S., Wendt-Potthoff K., Bhattacharya J. (2009). Occurrence and role of algae and fungi in acid mine drainage environment with special reference to metals and sulfate immobilization. Water Res..

[15] Hujslová M., Kubátová A., Kostovčík M., Blanchette R., Wilhelm de Beer Z., Chudíčková M., Kolařík M. (2014). Three new genera of fungi from extremely acidic soils. Mycol. Progress.

[16] Hujslová M., Bystrianský L., Benada O., Gryndler M. (2019). Fungi, a neglected component of acidophilic biofilms: Do they have a potential for biotechnology?. Extrem. Life Under Extrem. Cond..

[17] Oggerin M., Rodríguez M., del Moral C., Amils R. (2014). Fungal jarosite biomineralization in Río Tinto. Res. Microbiol..

[18] Marcos O., Zanettini J. (1982). Geología y Exploración del Proyecto Nevados del Famatina.

[19] Juarez O., Corbat M., Maza S., Collo G., Fucks E., Pannunzio E. (2019). Transformación Mineral de los Cementos de Ferricretes de la Formación Cueva de Pérez, Asociados a un Drenaje ácido Natural (Famatina, La Rioja). V Reunión Argentina de Geoquímica de la Superficie (RAGSU).

[20] Mackintosh M. (1978). Nitrogen Fixation by *Thiobacillus ferrooxidans*. Microbiology.

[21] Callahan B., McMurdie P., Rosen M., Han A., Johnson A., Holmes S. (2016). DADA2: High-resolution sample inference from Illumina amplicon data. Nat. Methods.

[22] McMurdie P., Holmes S. (2013). phyloseq: An R package for reproducible interactive analysis and graphics of microbiome census data. PLoS ONE.

[23] Martin-Pozas T., Fernandez-Cortes A., Cuezva S., Cañaveras J., Benavente D., Duarte E., Sanchez-Moral S. (2023). New insights into the structure, microbial diversity and ecology of yellow biofilms in a Paleolithic rock art cave (Pindal Cave, Asturias, Spain). Sci. Total Environ..

[24] Huang Y., Li X., Jiang Z., Liang Z., Wang P., Liu Z., Jiang C. (2021). Key factors governing microbial community in extremely acidic mine drainage (pH < 3). Front. Microbiol..

[25] Demir E., Yaman B., Çelik P., Sahinkaya E. (2020). Iron oxidation in a ceramic membrane bioreactor using acidophilic bacteria isolated from an acid mine drainage. J. Water Process Eng..

[26] Souza-Egipsy V., González-Toril E., Zettler E., Amaral-Zettler L., Aguilera A., Amils R. (2008). Prokaryotic community structure in algal photosynthetic biofilms from extreme acidic streams in Río Tinto (Huelva, Spain). Int. Microbiol..

[27] Gavrilov S., Korzhenkov A., Kublanov I., Bargiela R., Zamana L., Popova A., Golyshina O. (2019). Microbial communities of polymetallic deposits’ acidic ecosystems of continental climatic zone with high temperature contrasts. Front. Microbiol..

[28] Geremia R., Pușcaș M., Zinger L., Bonneville J., Choler P. (2016). Contrasting microbial biogeographical patterns between anthropogenic subalpine grasslands and natural alpine grasslands. New Phytol..

[29] de Mesquita C., Sartwell S., Ordemann E., Porazinska D., Farrer E., King A., Schmidt S. (2018). Patterns of root colonization by arbuscular mycorrhizal fungi and dark septate endophytes across a mostly unvegetated, high-elevation landscape. Fungal Ecol..

[30] Breitenbach R., Silbernagl D., Toepel J., Sturm H., Broughton W.J., Sassaki G., Gorbushina A. (2018). Corrosive extracellular polysaccharides of the rock-inhabiting model fungus *Knufia petricola*. Extremophiles.

[31] Zhao Y., Guo L., Xia Y., Zhuang X., Chu W. (2019). Isolation, identification of carotenoid-producing *Rhodotorula sp*. from marine environment and optimization for carotenoid production. Mar. Drugs.

[32] Li J., Jiang Z., Chen S., Wang T., Jiang L., Wang M., Li Z. (2019). Biochemical changes of polysaccharides and proteins within EPS under Pb (II) stress in *Rhodotorula mucilaginosa*. Ecotoxicol. Environ. Saf..

[33] Villanueva P., Vásquez G., Gil-Durán C., Oliva V., Díaz A., Henríquez M., Álvarez E., Laich F., Chávez R., Vaca I. (2021). Description of the First Four Species of the Genus *Pseudogymnoascus* from Antarctica. Front. Microbiol..

[34] Anastasi A., Spina F., Prigione V., Tigini V., Giansanti P., Varese G. (2010). Scale-up of a bioprocess for textile wastewater treatment using *Bjerkandera adusta*. Bioresour. Technol..

[35] Vázquez-Campos X., Kinsela A., Waite T., Collins R., Neilan B. (2014). *Fodinomyces uranophilus* gen. nov. sp. nov. and *Coniochaeta fodinicola* sp. nov., two uranium mine-inhabiting Ascomycota fungi from northern Australia. Mycologia.

[36] Hallberg K., González-Toril E., Johnson D. (2010). *Acidithiobacillus ferrivorans*, sp. nov.; facultatively anaerobic, psychrotolerant iron-, and sulfur-oxidizing acidophiles isolated from metal mine-impacted environments. Extremophiles.

[37] Barahona S., Castro-Severyn J., Dorador C., Saavedra C., Remonsellez F. (2020). Determinants of Copper Resistance in *Acidithiobacillus ferrivorans* ACH Isolated from the Chilean Altiplano. Genes.

[38] Liljeqvist M., Valdes J., Holmes D., Dopson M. (2011). Draft genome of the psychrotolerant acidophile *Acidithiobacillus ferrivorans* SS3. J. Bacteriol..

[39] Talla E., Hedrich S., Mangenot S., Ji B., Johnson D., Barbe V., Bonnefoy V. (2014). Insights into the pathways of iron-and sulfur-oxidation, and biofilm formation from the chemolithotrophic acidophile *Acidithiobacillus ferrivorans* CF27. Res. Microbiol..

[40] Hiraishi A., Imhoff J. (2015). Acidiphilium. Bergey’s Manual of Systematics of Archaea and Bacteria.

[41] Ullrich S., Poehlein A., Voget S., Hoppert M., Daniel R., Leimbach A., Mühling M. (2015). Permanent draft genome sequence of *Acidiphilium* sp. JA12-A1. Stand. Genom. Sci..

[42] Hu D., Cha G., Gao B. (2018). A phylogenomic and molecular markers based analysis of the class Acidimicrobiia. Front. Microbiol..

[43] Johnson D., Bacelar-Nicolau P., Okibe N., Thomas A., Hallberg K. (2009). *Ferrimicrobium acidiphilum* gen. nov., sp. nov. and *Ferrithrix thermotolerans* gen. nov., sp. nov.: Heterotrophic, iron-oxidizing, extremely acidophilic actinobacteria. Int. J. Syst. Evol. Microbiol..

[44] Liu Z., Liang Z., Zhou Z., Li L., Meng D., Li X., Yin H. (2021). Mobile genetic elements mediate the mixotrophic evolution of novel *Alicyclobacillus* species for acid mine drainage adaptation. Environ. Microbiol..

[45] Panyushkina A., Muravyov M., Fomchenko N. (2022). A Case of Predominance of *Alicyclobacillus tolerans* in Microbial Community during Bioleaching of Pentlandite-Chalcopyrite Concentrate. Minerals.

[46] Rowe O., Johnson D. (2008). Comparison of ferric iron generation by different species of acidophilic bacteria immobilized in packed-bed reactors. Syst. Appl. Microbiol..

[47] Mehrotra A., Sreekrishnan T. (2017). Heavy metal bioleaching and sludge stabilization in a single-stage reactor using indigenous acidophilic heterotrophs. Environ. Technol..

[48] Jones R., Johnson D. (2016). Iron kinetics and evolution of microbial populations in low-pH, ferrous iron-oxidizing bioreactors. Environ. Sci. Technol..

[49] Ma A., Zhuang X., Wu J., Cui M., Lv D., Liu C., Zhuang G. (2013). Ascomycota members dominate fungal communities during straw residue decomposition in arable soil. PLoS ONE.

[50] Méndez-García C., Peláez A., Mesa V., Sánchez J., Golyshina O., Ferrer M. (2015). Microbial diversity and metabolic networks in acid mine drainage habitats. Front. Microbiol..

[51] Aguilera A., González-Toril E., Tiquia-Arashiro S., Grube M. (2019). Eukaryotic Life in Extreme Environments: Acidophilic Fungi. Fungi in Extreme Environments: Ecological Role and Biotechnological Significance.

[52] Hujslová M., Gryndlerová H., Bystrianský L., Hršelová H., Gryndler M. (2020). Biofilm and planktonic microbial communities in highly acidic soil (pH < 3) in the Soos National Nature Reserve, Czech Republic. Extremophiles.

[53] Rousk J., Bååth E., Brookes P., Lauber C.L., Lozupone C., Caporaso J.G., Knight R., Fierer N. (2010). Soil bacterial and fungal communities across a pH gradient in an arable soil. ISME J..

[54] Stępniewska H., Uzarowicz Ł., Błońska E., Kwasowski W., Słodczyk Z., Gałka D., Hebda A. (2020). Fungal abundance and diversity as influenced by properties of Technosols developed from mine wastes containing iron sulphides: A case study from abandoned iron sulphide and uranium mine in Rudki, south-central Poland. Appl. Soil Ecol..

[55] Cai H., Shi P., Luo H., Bai Y., Huang H., Yang P., Yao B. (2011). Acidic β-mannanase from *Penicillium pinophilum* C1: Cloning, characterization and assessment of its potential for animal feed application. J. Biosci. Bioeng..

[56] López-Archilla A., González A., Terrón M., Amils R. (2004). Ecological study of the fungal populations of the acidic Tinto River in southwestern Spain. Can. J. Microbiol..

[57] Jin Y., Qin S., Gao H., Zhu G., Wang W., Zhu W., Wang Y. (2018). An anti-HBV anthraquinone from aciduric fungus *Penicillium sp*. OUCMDZ-4736 under low pH stress. Extremophiles.

[58] Dalsgaard P., Larsen T., Christophersen C. (2005). Bioactive cyclic peptides from the psychrotolerant fungus *Penicillium algidum*. J. Antibiot..

[59] Cantrell S., Dianese J., Fell J., Gunde-Cimerman N., Zalar P. (2011). Unusual fungal niches. Mycologia.

[60] Gomes E., Godinho V., Silva D., de Paula M.T.R., Vitoreli G.A., Zani C.L., Alves T.M.A., Junior P.A.S., Murta S.M.F., Barbosa E.C. (2018). Cultivable fungi present in Antarctic soils: Taxonomy, phylogeny, diversity, and bioprospecting of antiparasitic and herbicidal metabolites. Extremophiles.

[61] Martinez A., Cavello I., Garmendia G., Rufo C., Cavalitto S., Vero S. (2016). Yeasts from sub-Antarctic region: Biodiversity, enzymatic activities and their potential as oleaginous microorganisms. Extremophiles.

[62] Scardoni G., Laudanna C. (2012). Centralities Based Analysis of Complex Networks. New Frontiers in Graph Theory.

[63] Chung A., Lopes A., Nobre M., Morais P. (2010). *Hymenobacter perfusus* sp. nov., *Hymenobacter flocculans* sp. nov. and *Hymenobacter metalli* sp. nov. three new species isolated from an uranium mine waste water treatment system. Syst. Appl. Microbiol..

[64] Busarakam K., Bull A., Trujillo M., Riesco R., Sangal V., van Wezel G., Goodfellow M. (2016). *Modestobacter caceresii* sp. nov., novel actinobacteria with an insight into their adaptive mechanisms for survival in extreme hyper-arid Atacama Desert soils. Syst. Appl. Microbiol..

[65] Lynch R., King A., Farías M., Sowell P., Vitry C., Schmidt S. (2012). The potential for microbial life in the highest-elevation (>6000 masl) mineral soils of the Atacama region. J. Geophys. Res. Biogeosci..

[66] Ziegler S., Waidner B., Itoh T., Schumann P., Spring S., Gescher J. (2013). *Metallibacterium scheffleri* gen. nov., sp. nov., an alkalinizing gammaproteobacterium isolated from an acidic biofilm. Int. J. Syst. Evol. Microbiol..

[67] Zakharova K., Tesei D., Marzban G., Dijksterhuis J., Wyatt T., Sterflinger K. (2013). Microcolonial fungi on rocks: A life in constant drought?. Mycopathologia.

[68] Tesei D., Chiang A., Kalkum M., Stajich J., Mohan G., Sterflinger K., Venkateswaran K. (2021). Effects of simulated microgravity on the proteome and secretome of the polyextremotolerant black fungus *Knufia chersonesos*. Front. Genet..

