# Supplementary material for: The First Description of the Microbial Diversity in the Amarillo River (La Rioja, Argentina), a Natural Extreme Environment Where the Whole Microbial Community Paints the Landscape Yellow
Source: Microorganisms. 2024 Jan 23;12(2):235. doi: 10.3390/microorganisms12020235 (PMC10892261; doi:10.3390/microorganisms12020235)
Supplement: Supplementary file 1 [file microorganisms-12-00235-s001.zip › Supp File S1. Venn diagram fungal species.pdf]

Venn Diagram Fungi. Common ASVs and taxonomic affiliation according Silva database
ACP: Cueva de Perez terraces
AR: Amarillo River
FeEC: Fe(II) enrichment cultures

ACP-AR-FeEC
35 ASVs
Rhodotorula (2 ASVs), *Vishniacozyma*, *Naganishia*, *Bjerkandera*, unknown Fungi (2 ASVs), *Debaryomyces* (2 ASVs), *Coronicium*, *Microdochium*, *Sporothrix*, *Simplicillium*, *Leptobacillium*, unknown Sordariomycetes, *Cladosporium*, *Acrodontium*, *Fodinomyces*, *Verrucoconiothyrium*, *Alternaria*, *Knufia*, *Aspergillus*, *Penicillium* (5 ASVs), *Talaromyces* (2 ASVs), unknown Helotiales (2 ASVs), *Acidea*, unknown Ascomycota, *Acarospora*, *Talaromyces*, *Coniosporium*.

ACP-AR
16 ASVs
Rhodotorula, *Filobasidium*, unknown Fungi (2 ASVs), *Meyerozyma*, *Peroneutypa*, *Trichoderma*, unknown Capnodiales, unknown Chaetothyriales, *Peyronellaea*, *Leptospora*, unknown Pleosporales, *Aspergillus*, unknown Helotiales (2 ASVs), *Oidiodendron*

ACP-FeEC
21 ASVs
Hohenbuehelia, *Malassezia* (7 ASVs), *Kondoa*, *Phialemonium*, *Peltaster*, *Toxicocladosporium*, *Cladosporium*, *Mycosphaerella*, *Cladosporium*, unknown Fungi, *Neosulcatispora*, *Knufia*, *Aspergillus*, *Penicillium*, unknown Ramalinaceae, *Aureobasidium*

AR-FeEC
4 ASVs
Microsporomyces, *Acidea* (3 ASVs)
