# Supplementary figures and images for: The First Description of the Microbial Diversity in the Amarillo River (La Rioja, Argentina), a Natural Extreme Environment Where the Whole Microbial Community Paints the Landscape Yellow
Source: Microorganisms. 2024 Jan 23;12(2):235. doi: 10.3390/microorganisms12020235 (PMC10892261; doi:10.3390/microorganisms12020235)

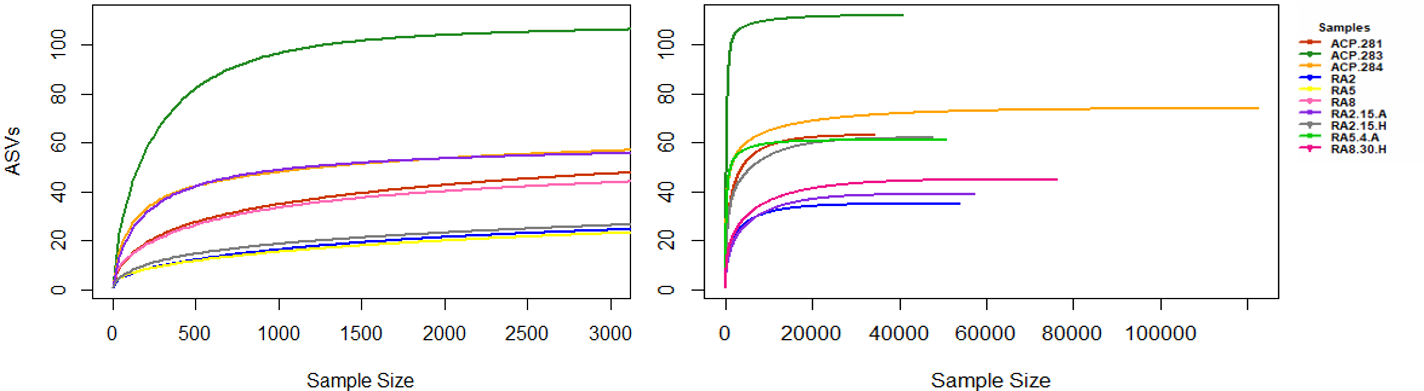

Supplement: Supplementary file 1 [file microorganisms-12-00235-s001.zip › Supp Figure S1. Rarefaction curves Bacteria and Fungi.png]
